# The Number of Overlapping AID Hotspots in Germline IGHV Genes Is Inversely Correlated with Mutation Frequency in Chronic Lymphocytic Leukemia

**DOI:** 10.1371/journal.pone.0167602

**Published:** 2017-01-26

**Authors:** Chaohui Yuan, Charles C. Chu, Xiao-Jie Yan, Davide Bagnara, Nicholas Chiorazzi, Thomas MacCarthy

**Affiliations:** 1 Department of Applied Mathematics and Statistics, Stony Brook University, NY, United States of America; 2 The Feinstein Institute for Medical Research, Northwell Health, Manhasset, NY, United States of America; 3 Departments of Medicine and Molecular Medicine, Hofstra Northwell School of Medicine, Hempstead, NY, United States of America; Institut de recherches cliniques de Montreal, CANADA

## Abstract

The targeting of mutations by Activation-Induced Deaminase (AID) is a key step in generating antibody diversity at the Immunoglobulin (Ig) loci but is also implicated in B-cell malignancies such as chronic lymphocytic leukemia (CLL). AID has previously been shown to preferentially deaminate WRC (W = A/T, R = A/G) hotspots. WGCW sites, which contain an overlapping WRC hotspot on both DNA strands, mutate at much higher frequency than single hotspots. Human Ig heavy chain (IGHV) genes differ in terms of WGCW numbers, ranging from 4 for IGHV3-48*03 to as many as 12 in IGHV1-69*01. An absence of V-region mutations in CLL patients (“IGHV unmutated”, or U-CLL) is associated with a poorer prognosis compared to “IGHV mutated” (M-CLL) patients. The reasons for this difference are still unclear, but it has been noted that particular IGHV genes associate with U-CLL vs M-CLL. For example, patients with IGHV1-69 clones tend to be U-CLL with a poor prognosis, whereas patients with IGHV3-30 tend to be M-CLL and have a better prognosis. Another distinctive feature of CLL is that ~30% of (mostly poor prognosis) patients can be classified into “stereotyped” subsets, each defined by HCDR3 similarity, suggesting selection, possibly for a self-antigen. We analyzed >1000 IGHV genes from CLL patients and found a highly significant statistical relationship between the number of WGCW hotspots in the germline V-region and the observed mutation frequency in patients. However, paradoxically, this correlation was inverse, with V-regions with more WGCW hotspots being less likely to be mutated, i.e., more likely to be U-CLL. The number of WGCW hotspots in particular, are more strongly correlated with mutation frequency than either non-overlapping (WRC) hotspots or more general models of mutability derived from somatic hypermutation data. Furthermore, this correlation is not observed in sequences from the B cell repertoires of normal individuals and those with autoimmune diseases.

## Introduction

Chronic lymphocytic leukemia (CLL) is the most common adult leukemia in the Western hemisphere. A key prognostic indicator for the disease is the mutational status of the Immunoglobulin heavy chain variable (IGHV) gene. An absence of significant numbers of mutations in the IGHV gene (<2% difference from germline) in CLL patients (“IGHV unmutated”, or U-CLL) is associated with a poorer prognosis compared to “IGHV mutated” (M-CLL) patients [1, 2]. The reasons for this difference are still unclear, but it has also been noted that particular IGHV genes and even particular IGHV alleles associate with U-CLL and others with M-CLL. For example, patients with IGHV1-69 clones have a strong tendency to be U-CLL and are associated with a poor prognosis [3], whereas patients with IGHV3-30 tend to be M-CLL and have a better prognosis, including some reported cases of spontaneous remission [4]. Furthermore, particular IGHV genes appear to fall outside of this categorization. For example, patients with IGHV3-21 clones tend to have a poor prognosis regardless of mutational status [5].

A distinctive feature of CLL is that ~30% of patients and ~50% of U-CLL, poor-outcome patients can be classified into a “stereotyped” subset, each defined by HCDR3 similarity, suggesting evidence for selection, possibly for a self-antigen [6]. Indeed, some candidate self-antigens have been identified, including non-muscle myosin heavy chain IIA [7], and cytoskeletal proteins such as vimentin [7, 8], filamin B and cofilin-1 [9]. Exogenous microbial (e.g. S. pneumoniae–[9, 10]) and viral (e.g. from herpesviruses such as Epstein-Barr or cytomegalovirus) antigens have also been implicated [11, 12]. Longer HCDR3s, which are also associated with poor prognosis in CLL [13], have a higher tendency for both self- and poly-reactivity [14, 15]. Interestingly, CLL cells do not survive or proliferate well *ex vivo* suggesting that the CLL microenvironment, which may facilitate antigen-mediated stimulation, is critical to disease progression [16].

For normal immune responses in B cells, the targeting of mutations caused by Activation-Induced Deaminase (AID) is a key step in generating antibody diversity at the Immunoglobulin loci, where it is involved in somatic hypermutation (SHM) of the variable (V) regions and class switch recombination (CSR) [17]. AID has previously been shown to preferentially deaminate WRC (W = A/T, R = A/G) hotspots both in vitro [18] and in vivo in the endogenous V region [19]. WGCW sites such as AGCT, which contain an overlapping WRC hotspot on both strands, tend to mutate at a far higher frequency than single WRC hotspots in V regions [20–22]. In switch regions there is a particularly high density of such sites, thus facilitating double-stranded breaks necessary for CSR [23]. More recent work has suggested that WGCW sites, and in particular certain AGCT sites may play a special role as AID “entry sites” that not only mutate at high frequency, but also facilitate further mutations close to the site of the original mutation and throughout the V region [24, 25]. In humans, IGHV genes differ greatly in terms of the number of WGCW hotspots, ranging from 4 for IGHV3-48*03 to as many as 12 in IGHV1-69*01.

Several recent studies have found that AID levels may be mis-regulated in CLL, particularly in U-CLL. For example, Palacios et al. found that high expression levels of AID were associated with increased cell proliferation and more active class-switching, as well as poor patient outcome [26]. A more recent study by Patten et al. extended these results showing that AID performs a full range of functions in CLL cells including somatic hypermutation, leading to substantial levels of intraclonal diversification [27]. However, in both studies it was clear that high AID expression was restricted to a small subset of cells, which are most likely proliferating, and that high AID expression was more frequently associated with U-CLL and poor patient outcome. It has been suggested that the association of AID expression with U-CLL may stem from the germline form of the Ig gene being optimal for antigenic drive, and thus AID-induced SHM may be selected against [28]. Chronically high levels of AID may in turn lead to increased risks, for both point mutations [29] and translocations [30]. The noncanonical Mismatch repair (MMR) pathway, which acts downstream of AID in normal somatic hypermutation, may also be disrupted in CLL, as it has been reported that Pol-η, a key component of this noncanonical MMR pathway, may be disregulated [31].

Here we analyzed >1000 heavy chain V-regions from CLL patients and found a highly significant statistical relationship between the number of WGCW hotspots in the germline V-region and the observed mutation frequency in patients. Paradoxically, the observed correlation is negative such that V-regions with more WGCW hotspots are less likely to be mutated, i.e., more likely to be U-CLL. We found that the correlation with WGCW hotspots is stronger than alternative measures such as total hotspots (overlapping and non-overlapping) or overall mutability. We discuss the possibility that a greater number of WGCW hotspots may be a hallmark of germline IGHV genes having a higher potential for self-reactivity.

## Materials and Methods

### Patients and IGHV sequence data

A total of 1158 subjects diagnosed with CLL enrolled in this study, which was approved by the Northwell Health Institutional Review Board. Subjects were selected based on diagnosis of CLL from our local CLL clinic and provided written informed consent to participate. A copy of the signed informed consent was given to each subject and the original signed documents remain securely stored at Northwell Health’s Feinstein Institute for Medical Research. After providing consent, peripheral blood samples and accompanying clinical data were obtained from each subject. From these samples, CLL clonal IGHV sequences were determined by direct sequencing of PCR products from cryopreserved peripheral blood mononuclear cells using primers previously described [32, 33] or by Cancer Genetics, Inc. (Rutherford, NJ). IGHV sequences were designated M-CLL if greater than 2% difference from germline sequences was determined by IMGT/V-QUEST [34]; otherwise they were designated as U-CLL.

### Bioinformatics analysis

All analyses were performed using custom R scripts. IGHV germline genes were obtained from IMGT [35]. In particular, the matchPattern function, part of the Biostrings library, was used to identify hotspot positions and regression analyses were performed using the lm function.

### Autoimmune disease sequence data

Autoimmune disease sequences were extracted from the NCBI Genbank database using the key words: “*rheumatoid arthritis; immunoglobulin; heavy; homo sapiens*”, “*multiple sclerosis; immunoglobulin; heavy; homo sapiens*”, and “*systemic lupus erythematosus; immunoglobulin; heavy; homo sapiens*”, respectively. All retrieved FASTA sequences were submitted to the IMGT High V-Quest webserver [36] using default settings. The alignments returned by IMGT were further filtered by the following criteria: 1) variable gene was identified; 2) V region identity > = 85%; 3) productive sequence. Clonally related sequences were then identified using the method of Chen et al. [37]. If clonally related sequences were identified, a single random sequence from each clonal group was chosen.

## Results

### The number of WGCW hotspots within germline IGHV genes is inversely correlated with mutation frequency in CLL

**Fig 1:**
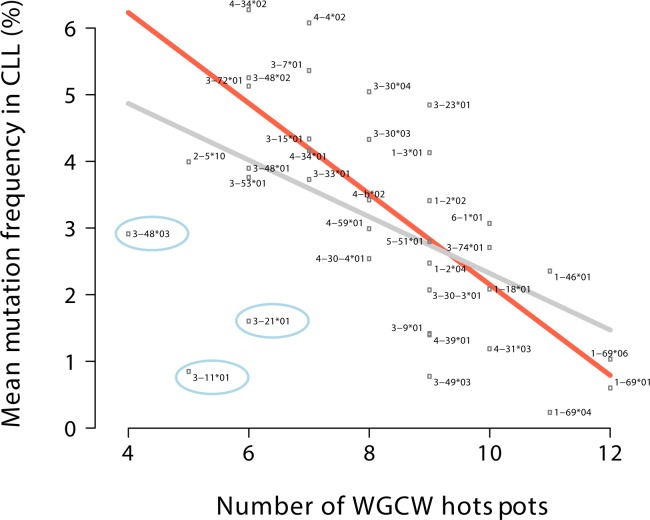
Comparison of the number of WGCW hotspots to mean mutation frequency. Number of WGCW hotspots in the germline IGHV gene sequence (horizontal axis) vs mean mutation frequency of each IGHV gene in CLL (vertical axis). Linear regression fit for all data points is shown by gray line; orange line shows fit with three outliers (encircled points) removed.

To investigate the relationship between AID overlapping hotspots and CLL mutation frequency, we grouped the CLL cases by V region gene and allele (e.g., IGHV3-23*01) and then calculated the mean mutation frequencies for each of these V regions. Our initial dataset is comprised of 1241 sequences from 1158 different CLL patients (see Methods). Because the variance of mean mutation frequency within each V region category can be high, we included in our analysis only V regions found in at least 10 CLL patients, leading to a total of 1002 sequences or >80% of the original 1241 patient sequences, with 36 different IGHV alleles represented. We compared the mean mutation frequency of each V gene to the number of overlapping AID hotspots (WGCW) in the corresponding germline V region, as identified by IMGT [34, 38]. Fig 1 shows the number of germline WGCW hotspots on the horizontal axis against the mean mutation frequency of each V gene on the vertical axis, with a linear regression fit shown by the gray line. The observed negative correlation is significant (Pearson r = -0.51, regression slope P = 1.1×10^−3^, R^2^ = 0.249) and paradoxical because V regions with more germline overlapping hotspots are less likely to be mutated in CLL. In other words, V regions with more WGCW hotspots are more likely to be U-CLL. For example, patients with IGHV1-69*01-expressing clones are usually unmutated CLL (U-CLL) and have a mean mutation frequency of 0.6%, whereas IGHV3-72*01-expressing clones are usually mutated and have a mean mutation frequency of 5.1%, an almost 10-fold difference. Surprisingly though, the IGHV1-69*01 germline sequence contains twice as many WGCW sites (12 overlapping hot spots) than IGHV3-72*01, which contains just 6 WGCW sites (Fig 1).

**Fig 2:**
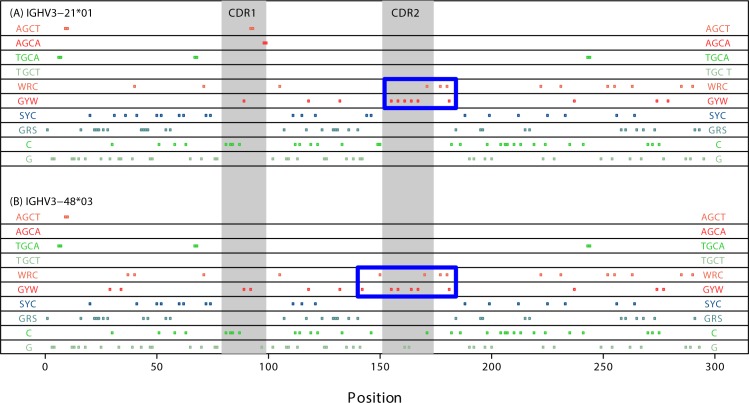
Profile of AID hotspots for (A) IGHV 3–21*01 and (B) IGHV3-48*03. Each colored dot in each panel represents an AID regular hot/cold/neutral spot as labeled at the edges. Double dots are overlapping hotspots (WGCW). CDR1 and CDR2 are indicated in gray shading. Although these genes have few WGCW hotspots, they both have a particularly dense region of regular GYW hotspots in CDR2, particularly so for IGHV 3–21*01. The blue boxes highlight dense clusters of non-overlapping WRC/GYW hotspots.

We further noted that the data contain three clear outliers: IGHV3-11*01, IGHV3-21*01 and IGHV3-48*03 (blue-encircled dots, Fig 1). If we exclude these three points from the regression, then the correlation is strengthened considerably (Fig 1 orange line, r = -0.76, regression slope P = 3.4×10^−7^, R^2^ = 0.560). To check that this result is not confounded by differential representation of the IGHV alleles in the cohort, we repeated the analysis using a weighted regression with the patient counts as weights and obtained almost identical results (P = 1.1×10^−7^, R^2^ = 0.589). One of the outlier genes, IGHV3-21, tends to have a poor prognosis regardless of mutational status [5], although recent analysis showed this may be due to the dominance of stereotyped subset #2 within these patients [39]. Interestingly, although IGHV3-21*01 has relatively few overlapping hotspots overall and none at all in CDR2, it does contain a tightly arranged cluster of 9 regular (i.e., non overlapping) WRC/GYW hotspots in CDR2 and the adjacent 5’ region of FW3 (blue box, Fig 2A). IGHV3-48*03 follows a very similar pattern (blue box, Fig 2B) but in addition has two WGCW sites less than the other alleles analyzed, IGHV3-48*01 and *02.

As an alternative test, we compared unmutated (U-CLL) to mutated (M-CLL) cases, as defined by the 2% mutation threshold that is usually used to discriminate U-CLL from M-CLL [1, 2]. Here we found that the number of AID overlapping hotspots in the germline V region of unmutated (U-CLL) cases is significantly higher than for mutated (M-CLL) cases (t-test, P<1×10^−15^). Because a large fraction (129/1002 = 12.9%) of cases in our dataset correspond to IGHV1-69 genes of which the majority (~90%) are U-CLL, we checked whether the observed difference remained if we removed the IGHV1-69 cases. Indeed, the difference in WGCW hotspots remained significant (P = 9.4×10^−4^). Thus, the number of WGCW hotspots is significantly higher in U-CLL vs M-CLL, and this difference is not due exclusively to the overabundance of IGHV1-69 cases.

**Fig 3:**
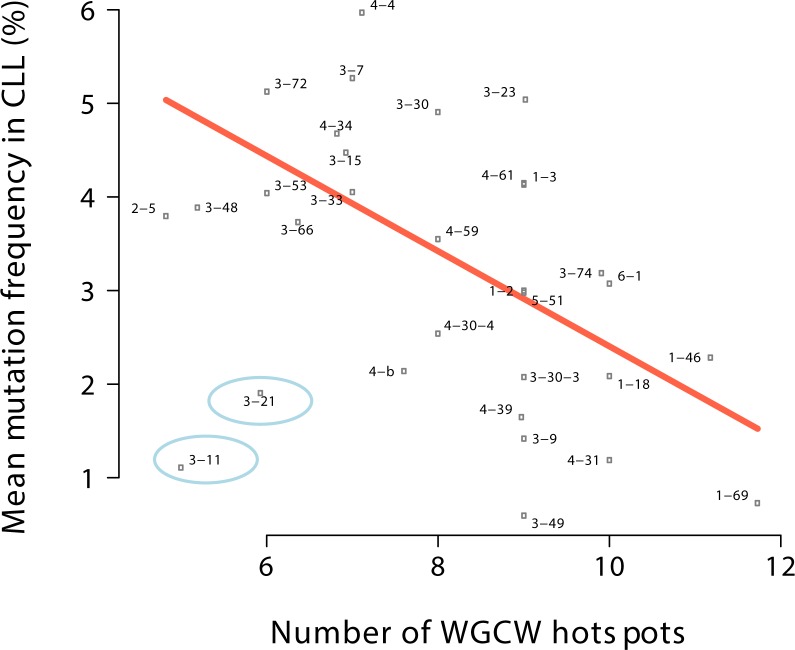
Comparison of the number of WGCW hotspots to mean mutation frequency aggregated by gene. This plot is equivalent to Fig 1, except that each point here is aggregated by gene (e.g. IGHV3-23) by combining all patients with IGHV3-23 alleles (e.g. IGHV3-23*01, IGHV3-23*02, etc.) into single data points. The regression line (orange) was fit excluding the outliers (encircled points).

We reevaluated all the data after combining all the alleles of each V gene together (e.g. IGHV3-23, IGHV4-34, and so on) rather than treating the alleles separately (e.g. IGHV3-23*01, IGHV3-23*02, and so on). Again we filtered out any cases that had fewer than 10 patients and removed two outliers (in this case only IGHV3-11 and 3–21). We similarly found a highly significant negative correlation (r = -0.6, regression slope P = 5×10^−4^, R^2^ = 0.342) as shown in Fig 3.

### Assessment of relationship with mutation frequency based on regular AID hotspots and mutabilities

We determined whether a relationship with mutation frequency might be stronger if we included all AID hotspots (WRC/GYW) and not only the overlapping (WCGW) sites. Unsurprisingly, given that WGCW sites are a subset of AID hotspots, we found that there was a significant negative correlation between the number of WRC/GYW hotspots and mutation frequency (for individual alleles, r = -0.49, regression slope P = 2.6×10^−3^; for genes, r = -0.44, P = 0.01), although the associated P values were weaker than for WGCW hotspots alone described above. To more formally assess the contribution of the WRC/GYW hotspots that are not overlapping WGCW sites, we evaluated a nested regression model that uses WGCW and non-overlapping WRC/GYW hotspots as independent variables, and compared this to a model that uses only WGCW. We found that non-overlapping WRC/GYW hotspots do not significantly improve the model regardless of whether the outliers are included (ANOVA, P = 0.20) or excluded (P = 0.81). We conclude that overlapping WGCW hotspots contain the essential signal and are therefore stronger negative predictors of mutation frequency in CLL than the more general motif for AID hotspots, WRC/GYW.

To further assess all positions, including A:T sites, we used previously published mutability scores [40]. These mutability scores are derived from silent mutations in several high-throughput somatic hypermutation datasets, and describe the relative mutational propensity for all nucleotide 5-mers, of which there are 4^5^ = 1024. We used the scores for all 5-mers in each V region, and then calculated their geometric mean to represent the overall mutability of the sequence. We found in this case that the correlations are negative, although the significance levels are marginal (for individual alleles, r = -0.36, P = 0.03; for genes, r = -0.41, P = 0.02) and the overall fit is poor (R^2^ = 0.1 and R^2^ = 0.14 for alleles and genes respectively). In conclusion, the count of WGCW hotspots for each V region appears to be a much stronger predictor of reduced mutation frequency in CLL than the more general features such as WRC/GYW and 5-mer mutability.

### WGCW hotspots are not predictive of mutation frequency in sequences from the B cell repertoires of normal individuals and those with autoimmune diseases

Having identified a negative correlation between WGCW hotspots and mutation frequency in CLL, we checked whether a similar relationship existed for V regions obtained from normal individuals. We used a previously published dataset of normal IGHV repertoire sequences [41] that include samples from spleen and peripheral blood. For each sample, B cells were sorted into subsets based on phenotype as follows: naïve (CD27-IgD+), marginal zone (CD27+IgD+), and memory (CD27+IgD-). IgM, IgA and IgG expression were further evaluated for the memory compartment. A single V region for each clonal group (as defined in [37]) was used to avoid possible double counting of clonally related mutations. As with the CLL sequences, only IGHV alleles with at least 10 sequences in the subset were used. In our analysis we were unable to identify a significant relationship, either positive or negative, between the number of WGCW hotspots and mutation frequencies in any of these subsets (S1 Table).

As mentioned in the Introduction, it has been suggested that clonal expansion of B cells in CLL, particularly at early stages of the disease, may be driven by self-antigen stimulation. We therefore analyzed three data sets of V regions from autoimmune disease samples that we obtained from GenBank (see Methods). These included samples from patients with Rheumatoid Arthritis (RA), Multiple Sclerosis (MS), and Systemic Lupus Erythematosus (SLE). In none of these cases did we find a significant relationship between the number of WGCW hotspots and mutation frequency (S2 Table) with the possible exception of RA, where we observe a marginally significant positive correlation (r = 0.42, P = 0.07, S1 Fig). We conclude that the negative correlation between WGCW hotspots and B-cell mutation frequency is observed for CLL clones only and not for V regions of clones from patients with certain autoimmune diseases or healthy individuals.

### No strong relationship between overlapping hotspots and clinical outcome

We analyzed the relationship between number of overlapping hotspots and clinical outcome, measured in two ways: time to first treatment (TTFT) and overall survival (OS). We first considered TTFT. Note that because only a subset of patients underwent treatment, we had fewer data points and we therefore reduced the cutoff for minimum number of patients from 10 to 4, although testing other cutoffs did not change the results qualitatively. We found no significant correlation between the number of WGCW sites in the germline IGHV and the mean TTFT for patients grouped either by IGHV allele (regression slope P = 0.49) or gene (P = 0.25). We repeated the analysis for OS and again found no significant correlations (by allele, P = 0.1; by gene, P = 0.1). Since the positive correlation between mutation frequency and clinical outcome is well established [1, 2] and because we found a negative correlation between the number of germline WGCW hotspots and mutation frequency as shown above, it is likely that with enough data we would indeed find a significant correlation between WGCW hotspots and clinical outcome. However, our results suggest the relationship between WGCW hotspots and clinical outcome is weaker.

### CDR3 length and the number of WGCW hotspots both show a negative correlation with mutation frequency, but are partially independent

Previous studies have reported a relationship in CLL between VH CDR3 length and mutation frequency, as well as clinical outcome (e.g. [13]). In concordance with previously reported results, our analysis shows that mean VH CDR3 length is negatively correlated with mutation frequency (r = -0.64, P = 2.96x10^-5^, R^2^ = 0.388, see S2 Fig). Thus less mutated (mostly U-CLL) V regions tend to be associated with longer CDR3s, a feature which in turn has been associated with a propensity for auto-reactivity [15]. To evaluate whether or not CDR3 length and the number of WGCW overlapping hotspots are independent predictors of mutation frequency in CLL, we compared regression models using only one of these variables (CDR3 length or the number of WGCWs) to models using both variables. We found in both cases that the two-variable regression model was a significant improvement over both single-variable models. Thus comparing the WGCW-only model (considering separate alleles and without the exclusion of outliers described above) with a model using both variables gives P = 6.7x10^-5^ (ANOVA), and the equivalent result for the CDR3-only model gives P = 0.018. These results further suggest that VH CDR3 length and the number of WGCW hotspots in the corresponding V segment are positively correlated, which is indeed the case (r = 0.37, P = 0.03, R^2^ = 0.111), although the relationship is not particularly strong.

### Analysis of potential for amino-acid changes at overlapping hotspot sites shows no difference between U and M-CLL

The analyses described above considered overlapping hotspot sites without taking into account the amino acid changes that might occur, and in particular whether these amino-acid changes might be different in U-CLL compared to M-CLL. To address this issue we first selected from the data points shown in Fig 1 (excluding the outliers), the 7 IGHV alleles having mean mutation frequency lower than 2% to represent U-CLL, and correspondingly selected the 7 alleles with the highest mutation frequency to represent M-CLL. For each of these sequences we identified the G and C sites of each WGCW motif and recorded the corresponding amino acid that would potentially be affected by a mutation, considering only replacement mutations. Note that overlapping hotspots affecting a Valine are always silent. This is because the 4 codons for Valine (defined by the motif GTN) can only be part of an overlapping hotspot if the TN dinucleotide matches TG in a TGCA or TGCT overlapping hotspot motif, and in this case the mutation in G will always be silent. Table 1 shows the counts of amino acids that can potentially mutate (columns U.total and M.total), the percentage these represent of the total (U.norm, M.norm) and whether they are in CDR or FW (U.FW, U.CDR, M.FW, M.CDR). We first compared the overall distribution (U.total vs M.total) and found there was no significant difference between U-CLL and M-CLL (n.s. using χ^2^ test and ignoring rows containing zeros in both U and M). For each amino-acid we also compared the balance between the number of hotspots in FW and CDR, and again found no significant difference for any amino acid (using the χ^2^ test). Lastly, it is noteworthy that there are so many overlapping hotspots affecting Serine. The AGC codon represents Serine and is also an AID hotspot. If the hotspot were non-overlapping, then a C>T mutation would be silent, but if the codon is part of an AGCT hotspot then a mutation in the bottom strand hotspot (G site) always causes an amino-acid replacement. As shown on Table 1, in the U-CLL group more potential changes affect Serine than any other amino-acid, and in turn most of these are in the CDRs, although the trend is similar for M-CLL and the difference between U and M is not significant.

**Table 1:** Potential amino acid changes at WGCW hotspots.

Amino-acid	Abbr	U.total	U.norm	U.FW	U.CDR	M.total	M.norm	M.FW	M.CDR
Ala	A	13	16.0%	4	9	10	18.9%	7	3
Cys	C	5	6.2%	5	0	0	0.0%	0	0
Ile	I	0	0.0%	0	0	0	0.0%	0	0
Leu	L	13	16.0%	8	5	9	17.0%	7	2
Met	M	1	1.2%	1	0	1	1.9%	1	0
Phe	F	0	0.0%	0	0	0	0.0%	0	0
Trp	W	0	0.0%	0	0	0	0.0%	0	0
Val	V	0	0.0%	0	0	0	0.0%	0	0
Gly	G	0	0.0%	0	0	0	0.0%	0	0
His	H	2	2.5%	2	0	1	1.9%	1	0
Pro	P	0	0.0%	0	0	0	0.0%	0	0
Ser	S	26	32.1%	5	21	15	28.3%	3	12
Thr	T	0	0.0%	0	0	0	0.0%	0	0
Tyr	Y	0	0.0%	0	0	0	0.0%	0	0
Arg	R	0	0.0%	0	0	0	0.0%	0	0
Asn	N	0	0.0%	0	0	0	0.0%	0	0
Asp	D	0	0.0%	0	0	0	0.0%	0	0
Gln	Q	16	19.8%	14	2	14	26.4%	8	6
Glu	E	3	3.7%	0	3	0	0.0%	0	0
Lys	K	2	2.5%	0	2	3	5.7%	0	3

Comparing 7 U-CLL with 7 M-CLL alleles, we show the potential amino acids that would be changed as a result of a mutation in either the G or C of the WGCW motif, considering only replacement mutations (columns U.total and M.total). Also shown is the percentage these represent of the total (columns U.norm and M.norm, which add up to 100%), and the breakdown as to whether the replacement mutations are in CDR or FW (U.FW, U.CDR, M.FW, M.CDR).

To summarize, in our analysis of the amino acids that have the potential to be mutated in WGCW hotspots, we found no evidence for particular amino acids being preferred in U-CLL compared to M-CLL alleles, nor did we observe a significant difference in the usage of FW vs CDR, although these results may be a consequence of the small counts involved (Table 1).

## Discussion

Our analysis showed that, in CLL, V-regions having more WGCW hotspots in the germline sequences are less likely to be mutated, i.e., more likely to be U-CLL. This relationship is not observed either in IGHV regions of B cells from normal individuals or from autoimmune diseases we analyzed. We found that this relationship is stronger for WGCW hotspots than for regular WRC/GYW hotspots and 5-mer mutabilities [40], and that the relationship is not clearly associated with stereotyped vs non-stereotyped subsets (data not shown). Our results appear paradoxical in the light of previous work by ourselves [21, 24] and others [25] showing that WGCW hotspots are intrinsically highly mutable and appear to drive additional mutations around these hotspots and throughout the V-region. However, these previous studies only considered a small number of distinct V regions. Our analysis of V region mutations from normal individuals (S1 Table) shows that a greater number of WGCW hotspots do not necessarily lead to a higher mutation frequency in normal B cells undergoing SHM. This observation is consistent with a recent study in mice where the VDJ region was replaced with a Sμ switch region and then evaluated for mutations. While the mouse Sμ switch region contains a high density of AGCT hotspots, this density varies within the switch region, which led the authors to define sub-regions reflecting sparse, intermediate and dense AGCT densities. Interestingly, across the sub-regions mutation frequency did not correlate with AGCT density, suggesting that AID targeting is efficient once a threshold density is reached, and that higher densities do not necessarily increase the mutation targeting frequency [42].

V regions containing many WGCW sites may be under strong negative selection during SHM. For example, WGCW hotspots preferentially create V region deletions, as observed in the non-productive alleles of a mouse model [25], which will most often lead to nonfunctional protein coding sequences. Also, GC B cells may only acquire one mutation per cell cycle [43]. If the mutation frequency is limited this way, then a greater number of WGCW hotspots may produce a greater diversity of mutations in terms of their distribution throughout the V region. If we were to assume that most mutations are deleterious to B-cell receptor (BCR) function (i.e., destabilize the Ig molecule) and very few mutations are beneficial (i.e., lead to higher affinity binding), then V regions with more WGCW hotspots may display an evolved high-risk / high-payoff strategy that explores a greater variety of mutations albeit with a lower probability of finding a beneficial one.

The fact that not even silent mutations are observed in U-CLL suggests there may be a more fundamental difference between U-CLL and M-CLL that is unrelated to negative selection at the BCR level. There may be, for example, key differences in terms of AID activity and/or how AID is targeted to the Ig loci. Thus, for example, AID can be differentially spliced, leading to different AID isoforms in M-CLL vs U-CLL and thus potential differences in AID activity and function [44]. It has also been suggested that some as-yet unidentified AID co-factor that is necessary for V region targeting, is not expressed [45]. Moreover, class switch recombination (CSR), which requires AID targeting to the switch regions (adjacent to the V), has been shown to occur *in vivo* in many U-CLL cases [46, 47], although here it might be argued that CSR and SHM are somewhat independent processes [48]. It should also be noted that any such differences between M-CLL and U-CLL are most likely dependent on the type of maturation that the CLL cell has undergone (T-cell dependent vs. T-cell independent [32]) and/or on the CLL microenvironment which would not be cell-intrinsic since outside of the *in vivo* disease context (*ex vivo* or in mouse xenograft) both types of CLL cells are capable of V region SHM, CSR and differentiation into plasma cells [27, 49].

It is possible that B cells containing IGHV genes with fewer WGCW hotspots target mutations more narrowly to particular sites, which in healthy individuals would usually be beneficial but which could potentially lead to auto-reactivity and M-CLL. Relevant to this point, a previous study [50] identified a limited subset of mutations that were highly recurrent in the V regions of certain stereotyped cases of M-CLL. Interestingly, several of these mutations occur in overlapping hotspots. For example, in IGHV4-34 stereotyped subsets #4 and #16, three particular codons (28, 40 and 45) were associated with mutations that were significantly “subset-biased” and of these, codon 40 occurs at an AGCT overlapping hotspot and is associated with a Serine to Threonine amino acid change. Similarly, for IGHV3-21 stereotyped subset #2, two of four sites identified are AGC Serine codons at overlapping AID hotspots, with codon 32 being within an AGCT motif and codon 34 within an AGCA, although in the case of IGHV3-21 the mutations are commonly seen in non-stereotyped CLL also [50].

Another possible, though not mutually exclusive, explanation for our results is that germline V regions with more WGCW hotspots may be more prone to code for self-reactivity, particularly in their unmutated form. For example, IGHV1-69, in addition to having many overlapping hotspots, has a highly hydrophobic CDR2 [51], a feature that facilitates its interaction with the gp41 and gp120 proteins of HIV and the E2 membrane fusion glycoprotein of HCV [52–54], and which may also explain its frequent usage in broadly-neutralizing antibodies (bnAbs) for influenza [51]. However, these features of IGHV1-69 may create disadvantages in the form of increased self- and poly-reactivity, for example, in the context of HCV infection, where anti-HCV IGHV1-69 antibodies have been shown to cross-react with antibodies encoded by other common IGHV genes such as IGHV3-23 and IGHV3-21 [55]. In the particular context of HIV, it is known that many bnAbs exhibit poly-reactivity [56] including at least two derived from IGHV1-69 where the corresponding self-antigens have been identified [57]. If B-cell lymphomas such as CLL arise as a consequence of antigenic drive as is widely believed, then this might explain why IGHV1-69 is found in a disproportionately high number of B-cell lymphomas [58]. Thus, the fact that IGHV1-69 has a high number of overlapping hotspots may be related with its tendency towards self- or poly-reactivity, since the gene may have evolved to mutate rapidly as a consequence of these inherent tendencies. The same principle may apply to other IGHV genes also.

In the context of CLL, unmutated BCR Immunoglobulins (which we show have more overlapping AID hotspots) tend to be both self- and poly-reactive, in contrast to mutated BCRs where these characteristics tend to be reduced [7, 59, 60]. It has previously been suggested that the poly- and self-reactivity of the CLL BCRs, together with the cell-cell interactions that occur within “proliferation centers” of the CLL tissue microenvironment, may synergize to provide survival and growth signals for the tumor [16]. In the case of U-CLL, these signals would be produced continually because of the absence of IGHV mutations, whereas for M-CLL, the presence of mutations might lead to minimal or no self-reactivity. This might also be the case for IGHV4-34, which is intrinsically autoreactive but in CLL is usually mutated [32, 50, 61], perhaps reflecting the need for SHM to reduce self-reactivity. As mentioned above, a critical AID co-factor might not be expressed in U-CLL cells because of the activation/maturation pathway these cells might follow (T-cell dependent vs. T-cell independent [32]), which would make the AID-expressing subset of lymphocytes unable to generate V region mutations but capable of CSR [45] and therefore continued interaction with an auto- (or exo-) antigen might drive the proliferative subsets of IgG-positive / AID-positive cells that are often observed in U-CLL [26].

In fact, it has long been recognized that somatic hypermutation (SHM) itself has the capacity to generate highly self-reactive antibodies [62]. This occurs not only in germinal centers (GCs) but also to some extent in extrafollicular niches [63], which may develop in the CLL microenvironment and support a non-classical type of SHM [49]. Within GCs, B cells will usually be eliminated or inactivated by further SHM if they are cross-reactive with self antigen as long as they are of adequate affinity and there is sufficient target self-antigen available within the GC [64]. Self-reactive BCRs can however be maintained through positive selection if they cross-react with foreign antigen. Recent work has shown that anergic self-reactive B-cells that cross-react with foreign antigen can undergo “redemption” through SHM such that they lose self-reactivity while maintaining high affinity to foreign antigen [65]. In summary, SHM is a key step in the elimination of self-reactive antibodies, and if particular V genes, such as IGHV1-69, are prone to self-reactivity (both *a priori* and when mutated), then having a greater number of WGCW hotspots may increase the number of ways that the V region can mutate away from self-reactivity. Thus, large numbers of WGCW hotspots in a germline V region may represent a signature and a potential escape mechanism for a gene with inherent self-reactivity. Presumably any disadvantages (e.g. potential for self-reactivity) are outweighed by the advantages of generating higher affinity antibodies to foreign antigen and, more generally, there may be a tradeoff between potential self-reactivity and neutralizing ability. This observation is perhaps not surprising given that pathogens are under evolutionary pressure to avoid detection by evolving protein sequences as similar as possible to those of the host [58], a phenomenon known as “molecular mimicry”.

## Supporting Information

S1 Fig Comparison of the number of WGCW hotspots to mean mutation frequency for the Rheumatoid Arthritis (RA) sample. (PDF)

S2 Fig Comparison of VH CDR3 length to mean mutation frequency. Plot of mean CDR3 length (horizontal axis) vs mean mutation frequency in CLL (vertical axis). Linear regression fit is shown by orange line.
(PDF)

S1 Table Correlation analysis for samples from normal patients. (PDF)

S2 Table Correlation analysis for autoimmune datasets. (PDF)

S1 Data Sequence data used for analysis. (ZIP)
