# Supplementary figures and images for: The Number of Overlapping AID Hotspots in Germline IGHV Genes Is Inversely Correlated with Mutation Frequency in Chronic Lymphocytic Leukemia
Source: PLoS One. 2017 Jan 26;12(1):e0167602. doi: 10.1371/journal.pone.0167602 (PMC5268644; doi:10.1371/journal.pone.0167602)

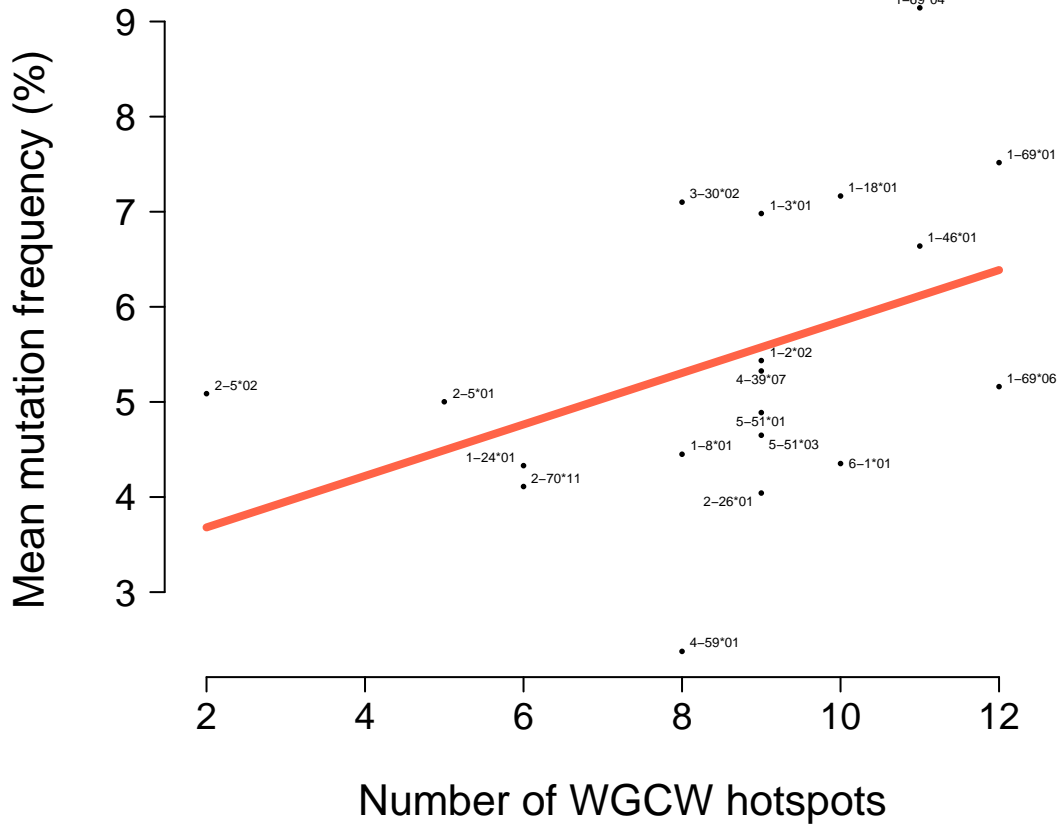

Supplement: S1 Fig — (PDF) [file pone.0167602.s001.pdf]

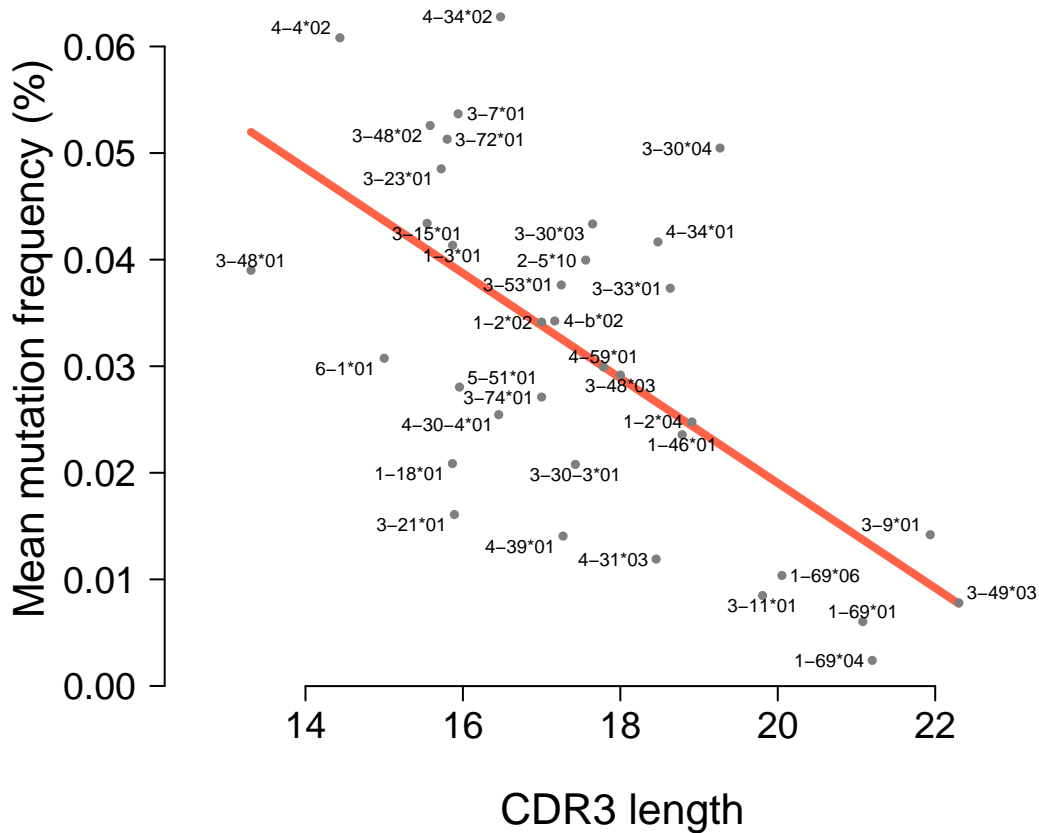

Supplement: S2 Fig — Plot of mean CDR3 length (horizontal axis) vs mean mutation frequency in CLL (vertical axis). Linear regression fit is shown by orange line. (PDF) [file pone.0167602.s002.pdf]
