# Supplementary material for: The Number of Overlapping AID Hotspots in Germline IGHV Genes Is Inversely Correlated with Mutation Frequency in Chronic Lymphocytic Leukemia
Source: PLoS One. 2017 Jan 26;12(1):e0167602. doi: 10.1371/journal.pone.0167602 (PMC5268644; doi:10.1371/journal.pone.0167602)
Supplement: S1 Table — (PDF) [file pone.0167602.s003.pdf]

**Cell type**	**Tissue**	**Ig class**	**N**	**correlation**	**P**
Marginal Zone	Blood	M	26	-0.316	0.116
Marginal Zone	Blood	M	18	0.119	0.638
Marginal Zone	Spleen	M	33	0.039	0.828
Marginal Zone	Spleen	M	21	-0.190	0.410
Naive	Blood	M	24	0.060	0.779
Naive	Blood	M	16	0.483	0.058
Naive	Spleen	M	27	-0.087	0.667
Naive	Spleen	M	18	0.225	0.369
Memory	Spleen	M	5	0.679	0.207
Memory	Spleen	M	5	0.679	0.207
Memory	Blood	A	8	-0.116	0.785
Memory	Blood	G	15	-0.327	0.234
Memory	Spleen	A	15	0.031	0.912
Memory	Spleen	G	21	0.336	0.136
