# Supplementary material for: The Number of Overlapping AID Hotspots in Germline IGHV Genes Is Inversely Correlated with Mutation Frequency in Chronic Lymphocytic Leukemia
Source: PLoS One. 2017 Jan 26;12(1):e0167602. doi: 10.1371/journal.pone.0167602 (PMC5268644; doi:10.1371/journal.pone.0167602)
Supplement: S2 Table — (PDF) [file pone.0167602.s004.pdf]

Condition	Grouping	Correlation	P
Rheumatoid_Arthritis	gene	0.418	0.075
Rheumatoid_Arthritis	allele	0.341	0.214
Multiple_Sclerosis	gene	-0.021	0.936
Multiple_Sclerosis	allele	0.240	0.323
Systemic_Lupus_Erythematosus	gene	0.125	0.875
Systemic_Lupus_Erythematosus	allele	0.182	0.730
